# Clinical Outcomes for *BRCA* Pathogenic Variant Carriers With Breast Cancer Undergoing Breast Conservation

**DOI:** 10.1001/jamanetworkopen.2024.18486

**Published:** 2024-06-25

**Authors:** Kerollos Nashat Wanis, Henry M. Kuerer, Susie X. Sun, Kelly K. Hunt, Alexa C. Glencer, Mediget Teshome, Anthony Lucci, Roi Weiser, Helen Johnson, Benjamin D. Smith, Angelica M. Gutierrez, Simona F. Shaitelman, Banu K. Arun

**Affiliations:** 1 Department of Breast Surgical Oncology, MD Anderson Cancer Center, Houston, Texas; 2 Department of Breast Radiation Oncology, MD Anderson Cancer Center, Houston, Texas; 3 Department of Breast Medical Oncology, MD Anderson Cancer Center, Houston, Texas

## Abstract

**Importance:**

Although most women with *BRCA-*associated breast cancer choose bilateral mastectomy, current guidelines support breast-conserving therapy as an option. As the indications for genetic testing expand and targeted therapies emerge, understanding the outcomes of breast-conserving therapy in the population of patients choosing breast conservation is important.

**Objective:**

To describe the clinical outcomes of women with *BRCA*-associated breast cancer who were treated with breast-conserving therapy, including the risks of ipsilateral and contralateral cancer events and bilateral mastectomy-free survival.

**Design, Setting, and Participants:**

This cohort study conducted at a single-institution academic national comprehensive cancer center included 172 women identified from a prospectively maintained database who had pathogenic *BRCA1/2* variants and were treated with breast-conserving therapy from January 1, 1977, to December 31, 2021.

**Main Outcomes and Measures:**

Clinical and pathologic characteristics for patients with *BRCA1* and *BRCA2* were compared, and estimates of overall survival, bilateral mastectomy-free survival, distant disease-free survival, risk of ipsilateral breast cancer, and risk of contralateral cancer were computed.

**Results:**

The cohort included 172 women (mean [SD] age, 47.1 [11.7] years), with 42 (24.4%) receiving a diagnosis of breast cancer prior to 40 years of age. Compared with *BRCA2* variant carriers (80 [46.5%]), women with *BRCA1* variants (92 [53.5%]) were younger at breast cancer diagnosis and tended to have more advanced tumors, which were more likely to be hormone receptor negative and higher grade. At a median follow-up of 11.8 years (IQR, 5.7-18.2 years), estimates of 10-year survival and risk were: overall survival, 88.5% (95% CI, 83.1%-94.2%); bilateral mastectomy-free survival, 70.7% (95% CI, 63.3%-78.9%); risk of an ipsilateral breast cancer event, 12.2% (95% CI, 5.8%-18.2%); and risk of contralateral cancer, 21.3% (95% CI, 13.3%-28.6%). Risks continued to increase after 10 years of follow-up.

**Conclusions and Relevance:**

In this cohort study, although women with breast cancer and pathogenic *BRCA1/2* variants treated with breast-conserving therapy had above-average risks of ipsilateral and contralateral breast cancer events, most did not have another cancer event and remained bilateral mastectomy free. These findings may be useful for informing patients with *BRCA* variants choosing breast conservation.

## Key Points

**Question:**

What are the long-term clinical outcomes of patients with *BRCA-*associated breast cancer who undergo breast-conserving therapy (BCT)?

**Findings:**

In this cohort study of 172 women with *BRCA-*associated breast cancer who underwent BCT, participants had above-average risks of ipsilateral and contralateral breast cancer events; however, if surviving to 10 years, most never experienced either event and were bilateral mastectomy free.

**Meaning:**

The long-term cancer event risks and the probability of future bilateral mastectomy can help inform patients with *BRCA-*associated breast cancer choosing breast conservation.

## Teaser

This cohort study describes the clinical outcomes of women with *BRCA1/2* variant–associated breast cancer who were treated with breast-conserving therapy, including the risk of ipsilateral and contralateral cancer events and bilateral mastectomy-free survival.

## Introduction

For patients with pathogenic *BRCA1/2* (*BRCA1*, OMIM 113705; *BRCA2*, OMIM 600185) variants, bilateral mastectomy is often used to reduce the risk of future breast cancers.^1,2^ Among patients who develop breast cancer in the context of a pathogenic *BRCA1/2* variant, bilateral mastectomy is very effective at reducing the risk of future contralateral breast cancers.^3^ In this setting, many patients elect to undergo bilateral mastectomy after diagnosis of breast cancer.^4,5^ But a smaller proportion of patients choose breast-conserving therapy (BCT), keeping their breasts and minimizing surgical morbidity^6^ while accepting a potentially higher future risk of ipsilateral and contralateral breast cancer events.^7^ Their choice is supported by existing guidelines that recommend, with moderate strength, that “germline *BRCA* status should not preclude a patient with newly diagnosed breast cancer otherwise eligible for BCT from receiving BCT.”^8^ This existing recommendation is based on evidence from observational studies, which have reported similar survival rates for BCT compared with mastectomy despite a higher risk of local cancer relapses (new primary cancers or recurrences) with BCT.^7,9,10,11^

More patients with breast cancer are undergoing germline *BRCA1/2* variant testing. Indications for testing have been expanded in recent guidelines, which now support testing in all patients with a new diagnosis of or with a personal history of breast cancer and for whom a poly(adenosine diphosphate-ribose) polymerase (PARP) inhibitor may be of benefit in decision-making about adjuvant systemic therapy.^12^ Among other benefits, identification of a *BRCA1/*2 variant supports enhanced surveillance imaging^13^ and allows patients to make informed decisions about treatment options, including emerging targeted therapies using PARP inhibitors, which have been shown to reduce recurrence among patients with *BRCA1* or *BRCA2* germline variant–associated early breast cancer.^14,15,16^ It is estimated that 5% to 10% of patients with breast cancer have a hereditary susceptibility and that pathogenic germline variants in *BRCA1/*2 account for one-third of these patients.^17^ Expanded testing will uncover many new cases.

Because a subset of patients with breast cancer and a pathogenic variant in *BRCA1/2* will choose BCT, continuing to study and report their long-term outcomes is important for ensuring that adequate evidence is available to inform patients. To that end, the objective of this study was to report the long-term clinical outcomes in a large, single-institution cohort of patients with pathogenic *BRCA1/*2 variants who chose BCT after initial diagnosis of breast cancer, with stratification of outcomes by *BRCA* variant type to help inform patients with a variant in either gene.

## Methods

### Study Population

We included patients with a pathogenic variant in *BRCA1/2* who had received a diagnosis of breast cancer and were treated with BCT. This cohort is nested within a University of Texas MD Anderson Cancer Center institutional review board–approved cohort of patients with a pathogenic variant in *BRCA1/2* seen by the Clinical Cancer Genetics Program at the University of Texas MD Anderson Cancer Center between January 1, 1997, and December 31, 2021. Patients provided written consent to collection of their clinical data within an institutional review board–approved prospective study. Patients were included regardless of the timing of their breast cancer diagnosis relative to the timing of their *BRCA1/2* variant diagnosis. We excluded patients who had received a diagnosis of breast cancer after 2021 due to inadequate length of follow-up and those with metastatic breast cancer at the time of diagnosis. This study followed the Strengthening the Reporting of Observational Studies in Epidemiology (STROBE) reporting guideline for cohort studies.^18^

### Variables

The MD Anderson Cancer Center prospectively managed Breast Cancer Management System Database was used, along with the electronic health record that was used to verify and update the recorded information on sociodemographic variables including self-reported race and ethnicity according to the electronic health record intake (assessed to describe the baseline characteristics of the cohort), sex, and age at diagnosis of breast cancer; clinical and tumor variables including timing of diagnosis relative to *BRCA1/2* variant diagnosis, history of ovarian cancer, history of bilateral salpingo-oophorectomy, menopausal status at breast cancer diagnosis, cancer laterality, cancer staging at diagnosis, tumor receptor status, tumor grade, and presence of lymphovascular invasion; and treatment variables describing surgical procedures, endocrine therapy, chemotherapy, and radiotherapy received over the duration of follow-up. Data on outcomes, including ipsilateral breast cancer events (local recurrences or new primary cancers), contralateral breast cancer, distant recurrence, and all-cause mortality, were reviewed and analyzed.

### Statistical Analysis

Patient, tumor, and treatment characteristics for the cohort of patients with a history of breast cancer treated with BCT and a known pathogenic variant in *BRCA1/2* were described. The distributions of characteristics for patients with *BRCA1* variants were contrasted with those for patients with *BRCA2* variants, and Fisher exact test *P* values were used to quantify the strength of the evidence for associations.

Overall survival, distant disease-free survival, and bilateral mastectomy-free survival, stratified by *BRCA* variant type (1 or 2), were estimated using the Kaplan-Meier estimator with loss to follow-up treated as a censoring event. The risk of ipsilateral breast tumor event (local recurrence or new ipsilateral cancer) and risk of contralateral breast cancer were estimated as the complement of their corresponding Kaplan-Meier survival estimates, with risk-reducing bilateral mastectomy treated as a censoring event. Last, the probabilities of survival without undergoing bilateral mastectomy and survival without undergoing bilateral mastectomy due to cancer, defined as bilateral mastectomy for an individual with a history of ipsilateral or contralateral breast cancer event, were computed. Death was treated as a censoring event for all outcomes except overall survival. Log-rank test *P* values were used to quantify the strength of the evidence for associations between *BRCA* variant type and outcomes.

To quantify the associations between patient, tumor, and treatment characteristics with ipsilateral breast cancer events and contralateral breast cancer, hazard ratios (HRs) were estimated using Cox proportional hazards regression models, and Wald test *P* values were used to quantify the strength of the evidence for conditional associations. The following characteristics were considered: type of *BRCA* variant, tumor hormone receptor status, stage at diagnosis, menopausal status at diagnosis, age at diagnosis, use of adjuvant endocrine therapy, use of chemotherapy, and bilateral salpingo-oophorectomy. Multivariable descriptive Cox proportional hazards regression models for ipsilateral breast cancer events and contralateral breast cancer, including all the previously listed characteristics, were fit to the data. Complete-case analysis was performed when missing data were present. Statistical analyses were performed using R, version 4.3.1 (R Project for Statistical Computing).

## Results

There were 172 women (mean [SD] age, 47.1 [11.7] years) treated with BCT who had pathogenic *BRCA* variants identified using the Clinical Cancer Genetics Program Database; 92 (53.5%) had *BRCA1* variants and 80 (46.5%) had *BRCA2* variants (Table 1). *BRCA* variants were detected before the first breast cancer diagnosis and treatment for 34 patients (19.8%) in the database. Patient, tumor, and treatment characteristics are presented in Table 1. Self-reported race for included patients was Asian for 10 patients (5.8%), Black for 26 patients (15.1%), Hispanic for 20 patients (11.6%), White for 115 patients (66.9%), and other for 1 patient (0.6%). Patients with *BRCA1* variants were more likely than those with *BRCA2* variants to receive a diagnosis of breast cancer prior to 40 years of age, were more likely to be premenopausal at breast cancer diagnosis, and tended to have more advanced primary tumors that were more likely to be hormone receptor negative and more likely to be high grade. Of the 42 patients (24.4%) who initially received a diagnosis of breast cancer prior to the age of 40 years, 6 (14.3%) became pregnant after BCT, and 4 (9.5%) had a live birth.

**Table 1.** Patient, Tumor, and Treatment Characteristics

Characteristic	No. (%) of patients			*P* value
	Overall (N = 172)	*BRCA1* variant (n = 92)	*BRCA2* variant (n = 80)	
Race and ethnicity				
Asian	10 (5.8)	2 (2.2)	8 (10.0)	.04
Black	26 (15.1)	15 (16.3)	11 (13.8)	
Hispanic	20 (11.6)	15 (16.3)	5 (6.2)	
White	115 (66.9)	59 (64.1)	56 (70.0)	
Other	1 (0.6)	1 (1.1)	0	
Sex				
Female	172 (100.0)	92 (100.0)	80 (100.0)	NA
Male	0	0	0	
Age at breast cancer diagnosis, y				
≥40	130 (75.6)	57 (62.0)	73 (91.2)	<.001
<40	42 (24.4)	35 (38.0)	7 (8.8)	
*BRCA* diagnosis known prior to initial BCT				
No	138 (80.2)	73 (79.3)	65 (81.2)	.85
Yes	34 (19.8)	19 (20.7)	15 (18.8)	
Ovarian cancer				
No	142 (82.6)	74 (80.4)	68 (85.0)	.55
Yes	30 (17.4)	18 (19.6)	12 (15.0)	
Ovarian cancer before breast cancer diagnosis				
No	163 (94.8)	85 (92.4)	78 (97.5)	.18
Yes	9 (5.2)	7 (7.6)	2 (2.5)	
Bilateral salpingo-oophorectomy				
No	52 (30.2)	28 (30.4)	24 (30.0)	>.99
Yes	120 (69.8)	64 (69.6)	56 (70.0)	
Bilateral salpingo-oophorectomy prior to breast cancer diagnosis				
No	148 (86.0)	79 (85.9)	69 (86.2)	>.99
Yes	24 (14.0)	13 (14.1)	11 (13.8)	
Menopause status at breast cancer diagnosis				
After	78 (45.3)	33 (35.9)	45 (56.2)	.009
Before	94 (54.7)	59 (64.1)	35 (43.8)	
Cancer laterality at diagnosis^a^				
Bilateral	7 (4.1)	4 (4.4)	3 (3.8)	>.99
Unilateral	164 (95.9)	87 (95.6)	77 (96.2)	
T category at diagnosis^b^				
T1	87 (52.4)	44 (50.0)	43 (55.1)	.09
T2	58 (34.9)	34 (38.6)	24 (30.8)	
T3	4 (2.4)	4 (4.5)	0	
Tis	17 (10.2)	6 (6.8)	11 (14.1)	
N category at diagnosis^c^				
N+	39 (23.8)	18 (20.9)	21 (26.9)	.46
N0	125 (76.2)	68 (79.1)	57 (73.1)	
Stage at diagnosis^d^				
0	16 (9.5)	5 (5.6)	11 (13.9)	.19
I	74 (44.0)	39 (43.8)	35 (44.3)	
II	67 (39.9)	37 (41.6)	30 (38.0)	
III	11 (6.5)	8 (9.0)	3 (3.8)	
Hormone receptor status^e^				
Negative	62 (47.3)	39 (58.2)	23 (35.9)	.01
Positive	69 (52.7)	28 (41.8)	41 (64.1)	
*ERBB2* status^f^				
Negative	113 (92.6)	58 (90.6)	55 (94.8)	.50
Positive	9 (7.4)	6 (9.4)	3 (5.2)	
Nuclear grade^g^				
I	5 (3.5)	3 (4.1)	2 (2.9)	.09
II	30 (21.1)	10 (13.7)	20 (29.0)	
III	107 (75.4)	60 (82.2)	47 (68.1)	
Lymphovascular invasion^h^				
Negative	130 (87.2)	72 (90.0)	58 (84.1)	.33
Positive	19 (12.8)	8 (10.0)	11 (15.9)	
Adjuvant endocrine therapy^c^				
No	97 (59.1)	66 (74.2)	31 (41.3)	<.001
Yes	67 (40.9)	23 (25.8)	44 (58.7)	
Chemotherapy^i^				
No	54 (32.1)	27 (29.7)	27 (35.1)	.51
Yes	114 (67.9)	64 (70.3)	50 (64.9)	
Radiotherapy^b^				
No	15 (9.0)	9 (9.9)	6 (8.0)	.79
Yes	151 (91.0)	82 (90.1)	69 (92.0)	

Abbreviations: BCT, breast-conserving therapy; NA, not applicable.

^a^ One participant with information not recorded.

^b^ Six participants with information not recorded.

^c^ Eight participants with information not recorded.

^d^ Four participants with information not recorded, but local or regional disease.

^e^ Forty-one participants with information not recorded.

^f^ Fifty participants with information not recorded.

^g^ Thirty participants with information not recorded.

^h^ Twenty-three participants with information not recorded.

^i^ Four participants with information not recorded.

Treatment included adjuvant radiotherapy for 151 of 166 patients (91.0%; 6 missing data), chemotherapy for 114 of 168 patients (67.9%; 4 missing data), and adjuvant endocrine therapy for 67 of 164 patients (40.9%; 8 missing data) (Table 1). None of the patients in the cohort experienced a radiotherapy-induced malignant neoplasm. Patients with *BRCA1* variants were less likely to receive treatment with adjuvant endocrine therapy. At the end of study follow-up for the current analysis, 120 patients (69.8%) had undergone a bilateral salpingo-oophorectomy, but only 24 (14.0%) had the procedure prior to their initial diagnosis of breast cancer.

### Survival and New Breast Cancer Events

**Figure 1.**
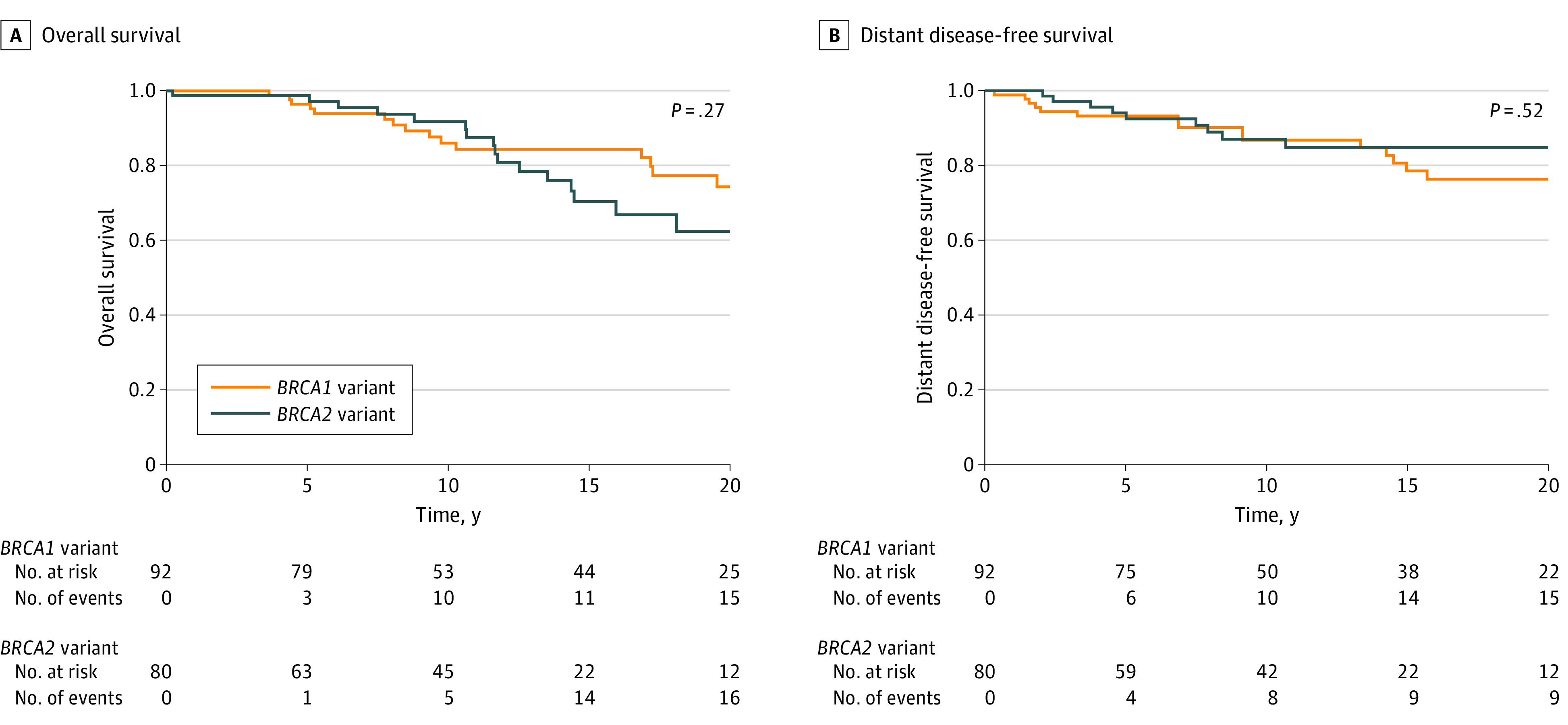
Overall Survival and Distant Disease-Free Survival Stratified by *BRCA* Variant Status Displayed *P* value is from log-rank test.

**Figure 2.**
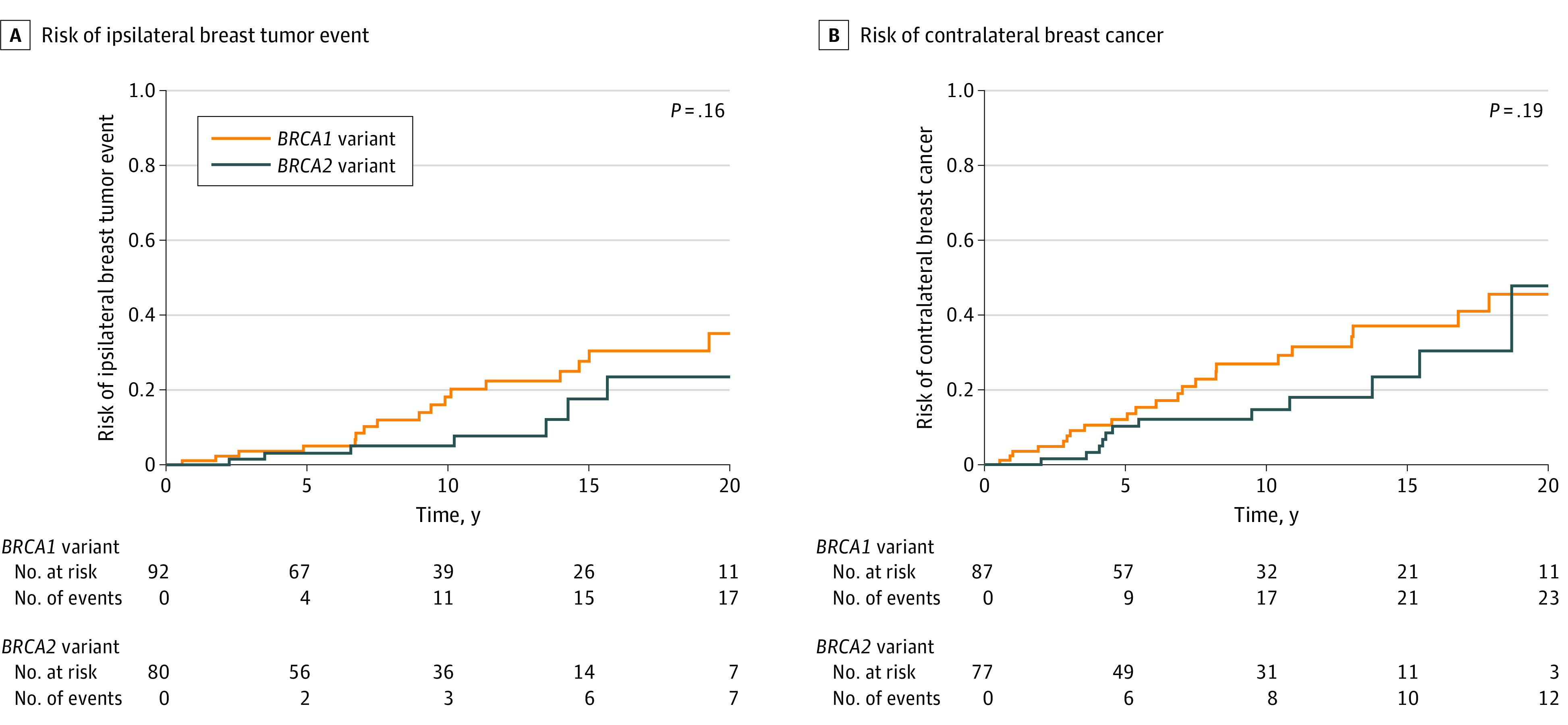
Risk of Ipsilateral Breast Tumor Event and Risk of Contralateral Breast Cancer Stratified by *BRCA* Variant Status Displayed *P* value is from log-rank test.

The cohort had a median follow-up time of 11.8 years (IQR, 5.7-18.2 years). At 10 years, the overall survival estimate was 88.5% (95% CI, 83.1%-94.2%), the distant disease-free survival estimate was 87.0% (95% CI, 81.4%-92.3%), the ipsilateral breast cancer event risk was 12.2% (95% CI, 5.8%-18.2%), and the contralateral breast cancer risk was 21.3% (95% CI, 13.3%-28.6%). Risks of ipsilateral and contralateral breast cancer events continued to increase at 20 years (Figure 1 and Figure 2). Of the 24 patients who experienced an ipsilateral breast tumor event, 3 patients (12.5%) had a second local recurrence event in the chest wall and received further treatment. Furthermore, 2 of those 3 patients had a third locally recurrent breast cancer event.

### Bilateral Mastectomies

**Figure 3.**
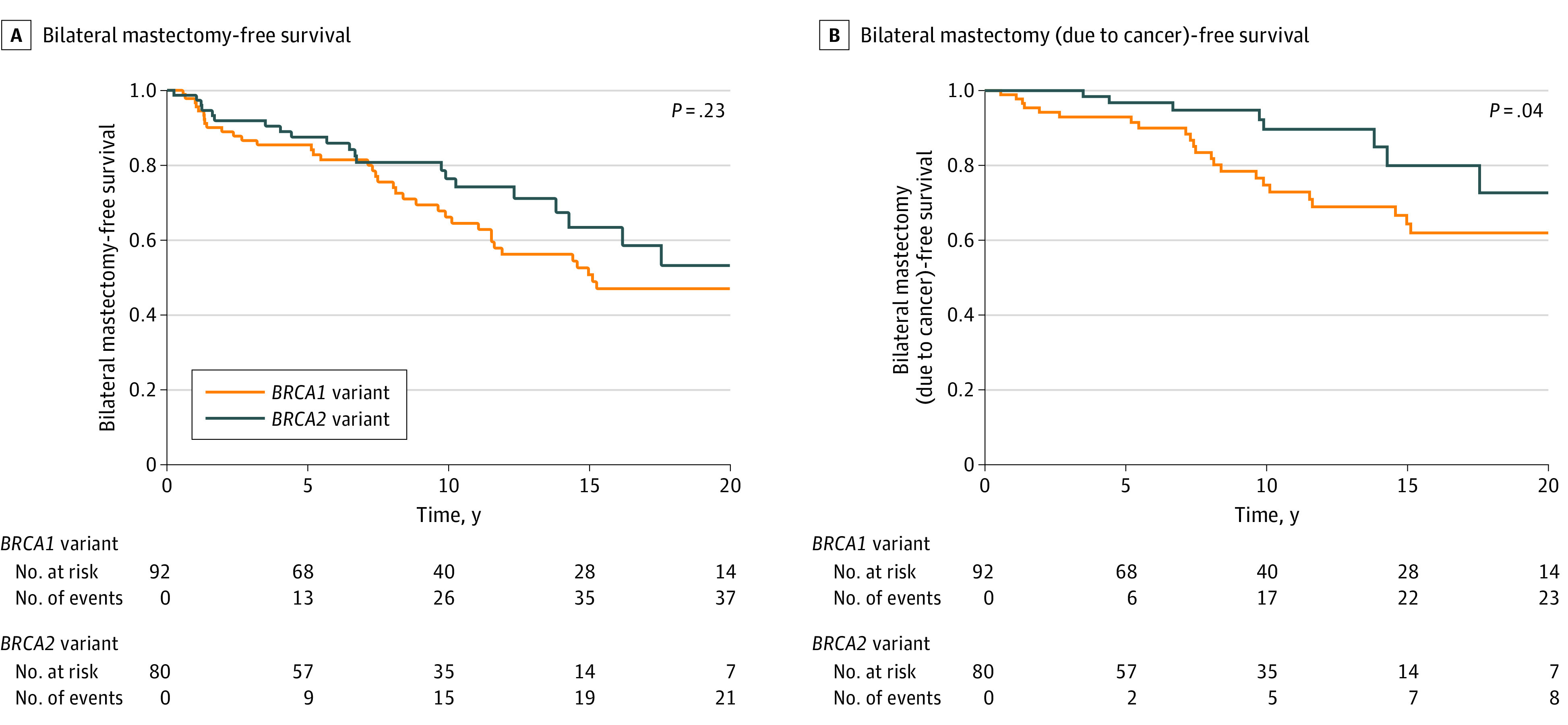
Bilateral Mastectomy-Free Survival and Bilateral Mastectomy-Free Survival Due to an Ipsilateral or Contralateral Breast Cancer Event Stratified by *BRCA* Variant Status Displayed *P* value is from log-rank test.

At 10 years, the bilateral mastectomy-free survival estimate was 70.7% (95% CI, 63.3%-78.9%) and the survival without bilateral mastectomy due to cancer estimate was 81.3% (95% CI, 74.4%-88.9%). The bilateral mastectomy-free survival curves, stratified by *BRCA1/2*, are shown in Figure 3. Half the participants ultimately underwent a bilateral mastectomy. Of 37 patients with *BRCA1* variants who underwent bilateral mastectomy, 14 (37.8%) did so in the absence of an ipsilateral or contralateral breast cancer event. In contrast, this number was 13 for the 21 patients (61.9%) with *BRCA2* variants.

### Patient, Tumor, and Treatment Characteristics Associated With Ipsilateral and Contralateral Breast Cancer Events

On univariate analysis, age younger than 40 years at initial breast cancer diagnosis (HR, 3.24 [95% CI, 1.43-7.32]) was positively associated with ipsilateral breast tumor events, and adjuvant radiotherapy (HR, 0.07 [95% CI, 0.02-0.28]) and bilateral salpingo-oophorectomy (HR, 0.33 [95% CI, 0.14-0.78]) were strongly inversely associated with ipsilateral breast tumor events (Table 2). In a multivariate model, adjuvant radiatiotherapy (HR, 0.09 [95% CI, 0.02-0.54]) and bilateral salpingo-oophorectomy (HR, 0.29 [95% CI, 0.09-0.90]) had strong inverse conditional associations with ipsilateral breast tumor events. The estimated HRs for all marginal (univariate) and conditional (multivariate) associations with ipsilateral and contralateral breast cancer event risk are presented in Table 2.

**Table 2.** HRs Quantifying the Marginal (Univariate) and Conditional (Multivariate) Associations Between Patient, Tumor, and Treatment Characteristics and Breast Cancer Events

Characteristic	Ipsilateral breast cancer events				Contralateral breast cancer events			
	Univariate		Multivariate		Univariate		Multivariate	
	HR (95% CI)	*P* value	HR (95% CI)	*P* value	HR (95% CI)	*P* value	HR (95% CI)	*P* value
Pathogenic *BRCA* variant								
* BRCA1*	1 [Reference]	.16	1 [Reference]	.34	1 [Reference]	.26	1 [Reference]	.15
* BRCA2*	0.53 (0.22-1.29)		1.92 (0.51-7.27)		0.70 (0.37-1.31)		0.51 (0.21-1.28)	
Hormone receptor status								
Negative	1 [Reference]	.22	1 [Reference]	.67	1 [Reference]	.81	1 [Reference]	.31
Positive	0.52 (0.18-1.48)		0.73 (0.18-3.04)		1.09 (0.52-2.30)		1.67 (0.63-4.44)	
Stage at diagnosis								
0/I	1 [Reference]	NA	1 [Reference]	NA	1 [Reference]	NA	1 [Reference]	NA
II	1.12 (0.47-2.63)	.80	2.26 (0.70-7.31)	.17	0.62 (0.32-1.20)	.16	0.87 (0.35-2.16)	.76
III	0.97 (0.21-4.37)	.96	1.72 (0.27-11.0)	.57	0.37 (0.09-1.58)	.18	0.60 (0.12-2.92)	.52
Menopause status								
After	1 [Reference]	.16	1 [Reference]	.98	1 [Reference]	.93	1 [Reference]	.85
Before	1.87 (0.77-4.51)		0.98 (0.23-4.13)		0.97 (0.53-1.78)		0.91 (0.34-2.41)	
Age, y								
≥40	1 [Reference]	.005	1 [Reference]	.15	1 [Reference]	.63	1 [Reference]	.68
<40	3.24 (1.43-7.32)		3.09 (0.68-14.1)		1.17 (0.61-2.24)		1.26 (0.42-3.74)	
Adjuvant endocrine therapy								
No	1 [Reference]	.08	1 [Reference]	.43	1 [Reference]	.30	1 [Reference]	.14
Yes	0.41 (0.15-1.11)		0.56 (0.13-2.38)		0.70 (0.36-1.36)		0.46 (0.16-1.29)	
Chemotherapy								
No	1 [Reference]	.22	1 [Reference]	.71	1 [Reference]	.10	1 [Reference]	.69
Yes	1.86 (0.69-4.99)		0.76 (0.18-3.27)		0.60 (0.33-1.11)		0.81 (0.28-2.28)	
Radiotherapy								
No	1 [Reference]	<.001	1 [Reference]	.008	1 [Reference]	.11	1 [Reference]	.52
Yes	0.07 (0.02-0.28)		0.09 (0.02-0.54)		0.37 (0.11-1.25)		0.59 (0.11-3.00)	
Bilateral salpingo-oophorectomy								
No	1 [Reference]	.01	1 [Reference]	.03	1 [Reference]	.25	1 [Reference]	.27
Yes	0.33 (0.14-0.78)		0.29 (0.09-0.90)		0.67 (0.34-1.33)		0.60 (0.24-1.47)	

Abbreviations: HR, hazard ratio; NA, not applicable.

## Discussion

In this study, the risks of ipsilateral and contralateral breast cancer events for patients with pathogenic *BRCA1/2* variants who were treated with BCT were higher than reported for the general breast cancer population. However, most patients did not have an ipsilateral breast cancer event, and half of the patients did not undergo bilateral mastectomy at 20 years from BCT. Younger patients were more likely to experience future ipsilateral breast cancer events, while adjuvant radiotherapy use and bilateral salpingo-oophorectomy were associated with a lower risk of ipsilateral breast cancer events.

The general breast cancer population risk of contralateral breast cancer for women with a diagnosis of unilateral cancer is less than 10% at 25 years from initial diagnosis.^19^ In contrast, we estimated the risk to be 21.3% at 10 years and nearly 50% at 20 years for patients with breast cancer who had a pathogenic *BRCA1/2* variant. This estimate is similar to that reported in prior cohorts.^20,21,22,23,24^ Basu et al^23^ reported that the risk of contralateral breast cancer in this population is 2% to 3% per year and is relatively constant for 20 years. This finding is consistent with our estimates and with a multi-institutional study by Graeser et al,^22^ who reported a 47.4% contralateral breast cancer risk at 25 years from initial cancer diagnosis. Kuchenbaecker et al^20^ estimated contralateral breast cancer risk at 20 years to be 40% for *BRCA1* variant carriers, but only 26% for *BRCA2* variant carriers. Metcalfe et al^25^ reported a 10-year risk of contralateral breast cancer of 43.4% for *BRCA1* carriers and 34.6% for *BRCA2* carriers who did not undergo oophorectomy or take tamoxifen. Our study similarly estimated that adjuvant endocrine therapy and history of oophorectomy were associated with lower risk of contralateral breast cancer, but the confidence intervals were wide. Evron et al^26^ studied contralateral prophylactic radiotherapy, finding that it reduces and delays the onset of contralateral breast cancers.

Ipsilateral breast cancer event risk is also reported to be higher for *BRCA1/2* variant carriers compared with the general population of patients treated with BCT for breast cancer. Nilsson et al^10^ estimated that the risk is 32% at 15 years, while Pierce et al^7^ reported it to be 30.2% at 20 years. These estimates are clearly higher than the generally accepted low risk of local failure for patients without *BRCA1/2* variants.^27^ These findings are further supported by a meta-analysis by Valachis et al,^28^ who reported a higher risk of ipsilateral breast cancer recurrence among variant carriers compared with noncarriers. However, some studies have reported conflicting findings. van den Broek et al,^9^ for example, reported nearly identical ipsilateral breast recurrence risks for variant carriers and noncarriers. Heterogeneity in the design of cohort studies of *BRCA1/2* variant carriers may explain some of these findings. Furthermore, treatment differences, including the use of adjuvant endocrine therapy, oophorectomy, adjuvant radiotherapy use, and adjuvant chemotherapy may dramatically affect the reported risks of local failure. We estimated that oophorectomy, adjuvant radiotherapy, and adjuvant endocrine therapy use were highly associated with lower risk of ipsilateral breast cancer events, although confidence intervals were wide for the latter.

Our finding that half the patients were bilateral mastectomy free at 20 years of follow-up may be very useful for shared decision-making discussions with patients. Although many patients who receive a diagnosis of breast cancer in the context of a *BRCA1/2* variant will choose bilateral mastectomy and, in doing so, reduce their risk of future breast cancer events, bilateral mastectomy has negative consequences on aspects of body image, and may be undesirable for some patients.^29,30^ For patients choosing breast conservation, one of the major themes underlying their decision is that keeping their breasts is “an important part of their feminine identity” and “central to feeling whole as a person.”^31^ Women who report that their breasts are very important to sexuality and the feeling of being feminine are more likely to choose breast conservation.^32^ For these women, bilateral mastectomy-free survival may be a relevant outcome because bilateral mastectomy has been reported to be associated with poorer sexual well-being and psychosocial well-being.^33^ Last, perioperative morbidity may be higher for mastectomy compared with BCT, which should be considered when coming to a shared decision about surgical approach.^34^

One aspect of this study that should be emphasized is that this is a particularly unique, large dataset of *BRCA* pathogenic variant carriers treated with breast-conserving surgery, with many of the patients included in this study being unaware of their *BRCA1/2* variant status at the time of their diagnosis and treatment for breast cancer. This allowed for a long-term study of the natural history of patients choosing BCT in the setting of a *BRCA* pathogenic variant, which was the core objective of this cohort study. We did not report outcomes of patients choosing bilateral mastectomy in this setting. Intractable confounding, particularly for overall survival, is likely in observational studies comparing the effectiveness of BCT and bilateral mastectomy in this population, and such a comparison was not the objective of this study.

### Limitations

This study has some limitations. Patients who have a *BRCA1/2* variant, but were unaware and never underwent testing (eg, because of a decision not to undergo testing or if they died from cancer or a noncancer cause prior to testing) would not have been included in this study. This selection induces immortal time, biasing survival estimates upward, if testing was not universal prior to death in our population, and may bias survival and cancer event risk estimates if receiving genetic testing is associated with outcomes. One way that this latter issue can manifest is in estimates of recurrence and contralateral breast cancer event risk that are higher than the true risk, if testing is more likely among patients who experience subsequent cancer events. Indeed, some guidelines support testing in such settings even though they do not recommend universal testing.^35^ van den Broek et al^9^ avoided this type of selection by testing all patients with breast cancer in their cohort, alive or deceased, using DNA from formalin-fixed paraffin-embedded normal tissue. They estimated that the 10-year risk of ipsilateral breast cancer events is 7.3%, suggesting that selection may have played a role in the high risks reported by studies similar to ours.^7,10^ The implication is that our estimates may be conservative. The downsides—future breast cancer events—for patients with *BRCA1/2* variants undergoing BCT may be overstated.

Beyond potential patient inclusion criteria issues affecting selection of patients into the study cohort, as in many other prospective clinical trials and institutional cohort studies, follow-up may have been truncated for patients who may be lost to follow-up or who relocated their care to another institution. To minimize the effect of this issue, patient outcomes were ascertained even when patients had aspects of their care managed at other institutions, and the cohort’s median length of follow-up was long (11.8 years). To reduce the risk of measurement error in any of the study variables, we manually reviewed all health care records contained within the institutional database for the cohort patients. Finally, some patients had BCT years before referral to our institution and, for a small proportion of these patients, some tumor factors were not recorded, as noted in Table 1.

## Conclusions

In this cohort study of BCT in pathogenic *BRCA1/2* variant carriers, we found that women with breast cancer and pathogenic variants in *BRCA1/2* treated with BCT have quantifiable above-average risks of ipsilateral and contralateral breast cancer events. However, most women in our cohort did not have another cancer event and half remained bilateral mastectomy free at 20 years. These findings may be useful for informing breast cancer patients who choose BCT, in the context of increasing testing and detection of pathogenic *BRCA* variants.

## Notes

Supplement.
Data Sharing Statement
