# Supplementary material for: Clinical Outcomes for BRCA Pathogenic Variant Carriers With Breast Cancer Undergoing Breast Conservation
Source: JAMA Netw Open. 2024 Jun 25;7(6):e2418486. doi: 10.1001/jamanetworkopen.2024.18486 (PMC11200147; doi:10.1001/jamanetworkopen.2024.18486)
Supplement: Supplement. — Data Sharing Statement [file jamanetwopen-e2418486-s001.pdf]

Data Sharing Statement

Wanis. Clinical Outcomes for BRCA Mutation Carriers With Breast Cancer Undergoing Breast Conservation. *JAMA Netw Open*. Published June 25, 2024. doi:10.1001/jamanetworkopen.2024.18486

Data
Data available: No

Additional Information
Explanation for why data not available: Patients did not consent to share their individual specific data.
